# Geographic differences in obesity prevalence and its risk factors among Asian Americans: findings from the 2013–2014 California Health Interview Survey

**DOI:** 10.1038/s41598-018-29906-5

**Published:** 2018-08-21

**Authors:** Shaoqing Gong, Kesheng Wang, Ying Li, Arsham Alamian

**Affiliations:** 1 Health Administration and Policy Institute, School of Public Policy and Administration, Xi’an Jiaotong University, Xi’an, Shaanxi Province China; 2 Department of Biostatistics and Epidemiology, College of Public Health, East Tennessee State University, Johnson City, Tennessee USA; 3 Department of Environment Health, College of Public Health, East Tennessee State University, Johnson City, Tennessee USA

## Abstract

Geography disparities exist in obesity and obesity related conditions. This study aimed to examine the geographic differences in obesity prevalence and its risk factors among Asian Americans in California. Data (n = 4,000) from the 2013–2014 California Health Interview Survey were used. Obesity (≥27.5 kg/m^2^) was defined according to the World Health Organization Asian body mass index cut points in Asian groups. Results suggest that 66.5% of Asians lived in urban areas. Among Asian adults, obesity prevalence was highest in Filipinos (33.8%) and lowest in Koreans (12.8%). Compared to rural Vietnamese, obesity prevalence was higher for urban Vietnamese (8.3% vs. 20.2%, p = 0.0318). Weighted multiple logistic regression analyses showed that being 45–64 years (vs. 65 years or above), being Japanese, Filipino, or other Asians (vs. Chinese) were associated with a higher odds of obesity among urban residents; whereas being 18–44 years and being 45–64 years (vs. 65 years or older), being male, having high school education (vs. having graduate education) were associated with a higher odds of obesity among rural residents. Being Vietnamese (vs. Chinese) was associated with 64% decreased odds of obesity only among rural residents (95% confidence interval = 0.14–0.94). The findings show geography disparities in obesity among Asians in California.

## Introduction

According to the National Health and Nutrition Examination Survey (NHANES) the age- adjusted prevalence of adult obesity was 37.7% in 2013–2014 in the United States (U.S.)^1^. Geography disparities exist in obesity and obesity related conditions. The burden of obesity is higher in the U.S. southern states than northern states^2^. About 70 million people live in rural areas, accounting for 23% of the U.S. population^3^. Compared with their urban counterparts, rural populations have higher rates of preventable conditions such as obesity and its related conditions (e.g., diabetes) as well as higher prevalence of all-cause mortality^4,5^; these adverse health differences observed in rural populations might be due to higher rates of health risk behaviors (e.g., smoking, physical inactivity, poor diet)^6–8^ and passive transportation means^9^ in rural areas. A recent study used measured height and weight in a nationally representative sample to examine the rural-urban differences in behavioral determinants of obesity and the independent effects of demographic and behavioral determinants among rural versus urban adults. The study concluded that obesity is markedly higher among rural adults than urban adults in the U.S. (39.6% versus 33.4%) and more attention is suggested to be paid to obesity in rural America^6^.

To date, there is very limited research on obesity among Asian Americans^10^. Compared to other ethnic groups (e.g., African Americans, Hispanic Americans), research on obesity in Asian Americans does not receive much attention, due to low overweight and obesity rates among them^10^. However, it has been suggested to use lower body mass index (BMI) cut points to define overweight and obesity among Asian Americans^11^, because Asian BMI cut points may provide better estimates of health conditions (e.g., diabetes) attributable to overweight and obesity^12–14^. Therefore, the prevalence of obesity reported by using BMI = 30 cut points for Asian Americans may be underestimated. The obesity definition using the WHO Asian BMI cut points (BMI = 27.5) in this study provided more accurate estimates of the actual prevalence. The present study is among the first studies to examine geography disparities in obesity prevalence in Asians in California.

The aims of this study were to 1) examine the geography (rural-urban) disparities in obesity prevalence among Asians and their subgroups (Chinese, Korean, Japanese, Filipinos, Vietnamese, and other Asians) in California; and 2) examine rural-urban differences in the risk factors of obesity among the Asian population of California.

## Methods

### Study population

The California Health Interview Survey (CHIS) is a random-digit-dial telephone survey of households designed to be representative of California’s noninstitutionalized population. A two-stage, geographically stratified design was used to produce a representative sample of the state. Residential telephone numbers were selected from within predefined geographic areas, and respondents were then randomly selected from within sampled households. The CHIS asks questions that are shared across age groups and also some questions that are unique to only one age group: children (0–11 years of age), adolescents (12–17 years of age), and adults (18 years and older). This study restricted the analysis to the Asian adults only (n = 4000). The telephone interviews allow the CHIS to track important health conditions and health behaviors in California. With a large sample of a diverse population, CHIS data have great ability to report on racial/ethnic differences.

The large CHIS sample includes people from many ethnic groups to provide health-related information for most large and small racial and ethnic populations that are all a part of California. To represent California’s diverse population and ensure that all ethnic groups can have a voice in representing the health of California, the CHIS is conducted in English, Spanish, Chinese (Cantonese and Mandarin dialects), Korean, Tagalog and Vietnamese. CHIS telephone surveys are conducted in all 58 counties of California. The CHIS may conduct oversampling of specific urban areas, such as Los Angeles and San Diego. In this study, data for years 2013 and 2014 were used for analyses. For CHIS 2013–2014, the landline/list sample household response rate was 14.8 percent; the cell sample household response rate was 16.6 percent.

The study obtained ethical approval from the institutional review boards of authors’ university. There was IRB exemption due to secondary data analysis.

## Study Variables

### Obesity

BMI was calculated using self-reported height and weight. In order to account for racial differences in body fat percentage at the same BMI level, we examined overweight and obesity using the WHO Asian BMI cut points in Asian groups as 4 categories: <18.5 kg/m^2^ (underweight), 18.5–22.9 kg/m^2^ (normal weight), 23–27.5 kg/m^2^ (overweight), and ≥27.5 kg/m^2^ (obesity)^15^.

### Geographical characteristics

A ZIP code-based geographic classification (Urban, 2^nd^ City, Suburban, and Town/Rural) was used to indicate geographic characteristics. Urban refers to ZIP codes associated with dense neighborhoods that represent the central cities of most major metropolitan areas (more than 4,150 persons/square mile). 2^nd^ City refers to ZIP codes associated with moderate-density neighborhoods in population centers (more than 1,000 and fewer than 4,150 persons/square mile). Suburban refers to ZIP codes associated with moderate-density neighborhoods that are not surrounded by urban or second-city population centers (estimated to be more than 1,000 persons/square mile and not in an urban or 2^nd^ city population center). Town/rural refers to ZIP codes associated with isolated small towns or less-developed areas on the exurban frontier (estimated to be more than 210 but fewer than 950 persons/square mile), or small villages and rural hamlets surrounded by productive farmland or wide-open spaces (estimated to be 210 or fewer persons/square mile). More details can be found elsewhere^16^.

### Covariates

Demographic variables included self-reported age, sex, and race/ethnicity (Asians: Chinese, Korean, Japanese, Filipino, Vietnamese, and other Asians). Family income was examined as a percentage of the federal poverty level (FPL). For FPL in 2013–2014, FPL cutoff points were obtained from the 2011 federal poverty guideline using the total household income and number of members in the household. Family income was categorized as below 100%, 100% to 299%, or 300% and above of the FPL. Smoking status was defined as current smoker or not current smoker. Marital status was defined as married, never married, or other. Education attainment included three categories including high school, college, and graduate. Physical activity was defined as walking at least 10 minutes for either transportation or leisure over the past 7 days. Fast food consumption was determined by response to the following question: “In the past 7 days, how many times did you eat fast food? Include fast food meals eaten at work, at home, or at fast-food restaurants, carryout or drive through” Response categories included: never, 1–2 times, and ≥3 times.

### Statistical analysis

Characteristics of California adults were examined by descriptive analysis according to geography, demographics, family income, weight status, and lifestyle risk factors. Obesity prevalence among adults was examined by geographic characteristics in 2013–2014. Variables with p values significant at 0.2 were included for analysis in adjusted models. SAS PROC SURVEYLOGISTIC procedure was used to estimate odds ratios (ORs) and 95% confidence intervals (CIs) for the association between independent variables and the prevalence of obesity. Multiple logistic regression analyses were used to examine the association between geographic characteristics, i.e., urban and rural (2^nd^ city, suburban, and town/rural) settings, and obesity after controlling for age, sex, race/ethnicity, family income, smoking status, marital status, education attainment, physical activity, and fast food consumption. Multiple logistic analyses were then used to examine the differences in risk factors for obesity in urban and rural settings, respectively.

Weights are included with the data files and are based on the State of California’s Department of Finance population estimates and projections, adjusted to remove the population living in group quarters (such as nursing homes, prisons, etc.) and thus not eligible to participate in CHIS. When the weights are applied to the data, the results represent California’s residential population during that year for the age group corresponding to the data file in use. Additional information on how to use the CHIS sampling weights, including sample code, is available at: http://healthpolicy.ucla.edu/chis/analyze/Pages/sample-code.aspx. All analyses were weighted to be representative of the California population and were adjusted for the complex survey design of the CHIS. All analyses were two-sided and performed with SAS version 9.4 (SAS Institute Inc, Cary, NC).

## Results

### Characteristics of Asian Americans by geographic location

Table 1 describes the participants’ characteristics of Asian Americans according to rural and urban areas. Overall, 66.5% of Asians lived in urban areas, 13.4% lived in a 2^nd^ city, 17.6% lived in suburban areas, and 2.5% lived in a town or rural areas. The majority of Asians were 18–44 years of age (55.8%), had high family income (54.4%, ≧300% FPL), were overweight or obese (63.9%), were not current smokers (91.2%), were married (54.5%), had college or graduate education degrees (72.5%), engaged in physical activity (81.1%), and consumed fast foods (56.1%). The largest percentage of Asian population consisted of Chinese (28.9%), followed by other Asians (20.7%), Filipinos (23.7%), Vietnamese (10.5%), Koreans (9.5%), and Japanese (6.7%). Urban Asians had higher percentage than rural Asians with regard to low education (i.e., high school degree, 30.1% vs. 22.4%, p = 0.0061) and poor family income (i.e., below 100% FPL, 15.3% vs.12.8%, p = 0.0003).

**Table 1:** Characteristics of Asian Americans by geographic location using CHIS 2013–2014.

	Overall (n = 4,000)	P-value	Rural* (n = 1,519)	Urban (n = 2,481)	P-value
	n (%)		n (%)	n (%)	
Geography characteristics		<0.0001			
Urban	2,481 (66.5)		NA	NA	
2^nd^ city	603 (13.4)		NA	NA	
Suburban	712 (17.6)		NA	NA	
Town or rural	204 (2.5)		NA	NA	
Age		<0.0001			0.7700
18–44 years	1,167 (55.8)		474 (57.0)	639 (55.3)	
45–64 years	1,523 (30.8)		621 (30.2)	902 (31.0)	
65 years or above	1,310 (13.4)		424 (12.8)	886 (13.7)	
Sex		<0.0001			0.3312
Male	1,751 (46.7)		654 (44.7)	1,097 (47.7)	
Female	2,249 (53.3)		865 (55.3)	1,384 (52.3)	
Asians		<0.0001			<0.0001
Chinese	1,128 (28.9)		376 (20.5)	752 (33.1)	
Korean	442 (9.5)		141 (10.0)	301 (9.2)	
Japanese	534 (6.7)		274 (10.0)	260 (5.1)	
Filipino	562 (23.7)		249 (28.7)	313 (21.2)	
Vietnamese	524 (10.5)		95 (6.2)	429 (12.7)	
Other	810 (20.7)		384 (24.7)	426 (18.7)	
Family income		<0.0001			0.0003
<100% FPL	770 (14.4)		190 (12.8)	580 (15.3)	
100–299% FPL	1,208 (31.1)		406 (25.2)	802 (34.1)	
≧300% FPL	2,022 (54.4)		923 (62.0)	1,099 (50.6)	
Weight status		<0.0001			0.5497
Underweight	132 (2.3)		48 (3.0)	84 (2.0)	
Healthy weight	1,406 (33.9)		485 (35.2)	921 (33.2)	
Overweight	1,595 (39.8)		601 (38.2)	994 (40.6)	
Obesity	867 (24.1)		385 (23.6)	482 (24.3)	
Smoking status		<0.0001			0.0352
Current smoker	266 (8.8)		95 (6.5)	171 (9.9)	
Not current smoker	3,734 (91.2)		1,424 (93.5)	2,310 (90.1)	
Marital status		<0.0001			0.8358
Married	2,324 (54.5)		913 (53.7)	1,411 (54.9)	
Others	885 (12.9)		322 (12.5)	563 (13.1)	
Never married	791 (32.6)		284 (33.8)	507 (32.0)	
Education		<0.0001			0.0061
High school	1,137 (27.5)		345 (22.4)	792 (30.1)	
College	2,005 (52.3)		782 (53.4)	1,223 (51.8)	
Graduate	858 (20.2)		392 (24.2)	466 (18.2)	
Physical activity		<0.0001			0.7654
No	806 (18.9)		332 (18.5)	474 (19.1)	
Yes	3,194 (81.1)		1,187 (81.5)	2,007 (80.9)	
Fast food consumption		<0.0001			0.5581
Never	2,072 (43.9)		715 (42.2)	1,357 (44.8)	
1–2 times	1,415 (37.1)		585 (39.0)	830 (36.1)	
≥3 times	513 (19.0)		219 (18.8)	294 (19.1)	

Abbreviation: FPL = federal poverty level.

*Rural refers to 2^nd^ City, suburban, town or rural.

### Percent of obesity by selected demographic characteristics in rural and urban Asian adults

Table 2 shows percent of obesity by selected demographic characteristics in rural and urban Asian adults in California. Among Asian adults, the prevalence of obesity was highest in Filipinos (33.8%), followed by Japanese (32.0%), other Asians (25.9%), Vietnamese (17.9%), Chinese (17.3%), and Koreans (12.8%). The rural-urban difference in obesity prevalence was only observed in Vietnamese, with higher rate in urban than rural residents (20.2% vs. 8.3%, p = 0.0318).

**Table 2:** Prevalence of obesity by selected demographic characteristics in rural and urban Asian adults in California.

Characteristics	Overall n (%)	Rural n (%)	Urban n (%)		p-value*
Race/ethnicity					
Asians					
Chinese	180 (17.3)	70 (19.3)	110 (16.6)	0.4400	
Korean	60 (12.8)	20 (11.4)	40 (13.6)	0.6961	
Japanese	177 (32.0)	94 (25.8)	83 (38.0)	0.1263	
Filipino	197 (33.8)	92 (30.6)	105 (35.9)	0.4025	
Vietnamese	73 (17.9)	13 (8.3)	60 (20.2)	0.0318	
Other	297 (25.9)	157 (26.5)	140 (25.4)	0.8182	
Gender					
Male	424 (28.4)	199 (30.0)	225 (27.7)	0.5286	
Female	443 (20.2)	186 (18.5)	257 (21.1)	0.4189	
Family income					
<100% FPL	133 (19.3)	42 (15.8)	91 (20.8)	0.3826	
100–299% FPL	266 (26.1)	114 (23.3)	152 (27.2)	0.4279	
≧300% FPL	468 (24.1)	229 (25.4)	239 (23.3)	0.4985	
Education					
High school	230 (22.6)	89 (28.4)	141 (20.4)	0.0899	
College	474 (26.7)	214 (23.6)	260 (28.3)	0.1733	
Graduate	163 (19.2)	82 (19.2)	81 (19.3)	0.9867	

Abbreviation: FPL = federal poverty level.

*p-value is calculated based on chi-square test.

### Multivariable analysis of obesity and its risk factors by geographic location

Table 3 shows the results of logistic regression analysis for the association between risk factors and obesity (vs. absence of obesity) among Asian Americans in California, overall and according to geography characteristics, respectively. Weighted multiple logistic regression analyses show that, overall, being 45–64 years (vs. 65 years or above) (OR = 1.98, 95% CI = 1.37–2.88), being male (OR = 1.56, 95% CI = 1.14–2.14), being Japanese (OR = 2.44, 95% CI = 1.53–3.91), Filipino (OR = 2.50, 95% CI = 1.66–3.75), or other Asians (OR = 1.82. 95% CI = 1.19–2.78) (vs. Chinese) were associated with higher odds of obesity. In urban areas, being 45–64 years (vs. 65 years or above) (OR = 2.04, 95% CI = 1.23–3.38), being Japanese (OR = 3.49, 95% CI = 1.84–6.62), Filipino (OR = 2.63, 95% CI = 1.59–4.36), or other Asians (OR = 1.93, 95% CI = 1.09–3.42) (vs. Chinese) were associated with a higher odds of obesity; whereas in rural areas, being 18–44 years (OR = 2.15, 95% CI = 1.14–4.06), being 45–64 years (OR = 1.94, 95% CI = 1.05–3.57) (vs. 65 years or above), being male (OR = 2.05, 95% CI = 1.27–3.29), and having high school education (vs. graduate education) (OR = 2.58, 95% CI = 1.19–5.61) were associated with a higher odds of obesity. In addition, being Vietnamese (vs. Chinese) (OR = 2.73, 95% CI = 1.35–5.51) was associated with decreased odds of obesity only in rural areas.

**Table 3:** Logistic regression analyses for the association between obesity (vs. absence of obesity) and its risk factors for the overall sample and urban and rural samples of Asian Americans in California, 2013–2014.

Characteristics	Overall (n = 4,000)	Urban (n = 1,519)	Rural^#^ (n = 2,481)
	AOR (95% CI)	AOR (95% CI)	AOR (95% CI)
Geography			
Urban (ref)			
Rural	0.88 (0.66–1.18)	N/A	N/A
Age			
65 years or above (ref)			
18–44 years	1.51 (0.98–2.34)	1.32 (0.71–2.47)	2.15 (1.14–4.06)*
45–64 years	1.98 (1.37–2.88)**	2.04 (1.23–3.38)*	1.94 (1.05–3.57)*
Sex			
Female (ref)			
Male	1.56 (1.14–2.14)*	1.37 (0.92–2.07)	2.05 (1.27–3.29)*
Race/ethnicity			
Chinese (ref)			
Korean	0.67 (0.37–1.22)	0.76 (0.34–1.70)	0.45 (0.19–1.08)
Japanese	2.44 (1.53–3.91)**	3.49 (1.84–6.62)**	1.23 (0.62–2.45)
Filipino	2.50 (1.66–3.75)***	2.63 (1.59–4.36)**	2.02 (0.97–4.20)
Vietnamese	1.11 (0.62–2.00)	1.36 (0.70–2.66)	0.36 (0.14–0.94)*
Other Asians	1.82 (1.19–2.78)*	1.93 (1.09–3.42)*	1.34 (0.75–2.38)
Family income			
≧300% FPL (ref)			
<100% FPL	0.86 (0.54–1.37)	1.03 (0.57–1.87)	0.55 (0.26–1.20)
100–299% FPL	1.23 (0.89–1.71)	1.35 (0.88–2.07)	0.94 (0.56–1.59)
Smoking status			
Not current smoker (ref)			
Current smoker	1.06 (0.65–1.74)	1.36 (0.78–2.36)	0.46 (0.17–1.24)
Marital status			
Married (ref)			
Never married	0.97 (0.64–1.47)	1.17 (0.67–2.03)	0.54 (0.27–1.09)
Others	1.14 (0.75–1.74)	1.17 (0.67–2.04)	1.10 (0.56–2.17)
Education			
Graduate (ref)			
High school	1.32 (0.79–2.19)	1.01 (0.51–2.00)	2.58 (1.19–5.61)*
College	1.29 (0.84–2.00)	1.26 (0.69–2.30)	1.34 (0.66–2.73)
Physical activity			
No (ref)			
Yes	0.82 (0.57–1.19)	0.84 (0.53–1.33)	0.85 (0.52–1.40)
Fast food consumption			
Never (ref)			
1–2 times	0.98 (0.71–1.35)	0.84 (0.53–1.33)	1.41 (0.85–2.32)
≥3 times	1.19 (0.76–1.87)	1.17 (0.64–2.14)	1.28 (0.62–2.61)

Abbreviations: AOR, adjusted odd ratio; CI, confidence interval; PFL, poverty federal level.

^#^Rural refers to 2^nd^ City, Suburban, and Town or Rural.

*P < 0.05, **P < 0.001, ***P < 0.0001.

## Discussion

The major finding of this study is the significantly higher prevalence of obesity in urban Vietnamese compared to rural Vietnamese adults in California. Rural-urban differences in obesity prevalence were not observed in other Asian subgroups. Rural residence was not found to be associated with higher obesity prevalence after controlling for covariates. Rural-urban differences were found in risk factors of obesity, e.g., being Vietnamese, being male, or less education was associated with obesity only among rural residents, whereas being Japanese, Filipino, or other Asians was associated with obesity only among urban residents. Based on our knowledge, this is among the first studies comparing rural and urban obesity prevalence using WHO Asian BMI weight status classification.

Many of the studies examining rural and urban differences in health outcomes use national surveillance data such as the National Health and Nutrition Examination Survey (NHANES), Behavior Risk Factor Surveillance System (YRBSS), and National Health Interview Survey (NHIS)^2,6^. Previous studies reported a significant rural-urban difference in obesity using self-reported data from NHIS 1997–1998 and BRFSS 2000–2001 (20–23% and 18–20% for rural and urban, respectively)^2,17^. Using NHANES data, Befort *et al*.^6^ showed that the obesity prevalence was 39.6% among rural adults compared to 33.4% among urban adults, and it remained significantly higher among rural compared to urban adults after adjusting for covariates (i.e., demographic, diet, and physical activity variables) (OR = 1.18)^6^.

Race/ethnicity and percent kilocalories from fat were significant correlates of obesity among both rural and urban adults. Being married was associated with obesity only among rural residents, whereas older age, less education, and being inactive was associated with obesity only among urban residents. Befort *et al*. concluded that obesity is markedly higher among adults from rural versus urban areas of the United States^6^. Reasons for the geographic health disparities include rural-urban differences in obesity in these studies, e.g., rural people tend to have higher rates than urban people for health risk behaviors (e.g., smoking, physical inactivity, poor diet)^6–8^ and passive transportation means^9^, which likely contribute to having higher obesity rates. The results of these studies are not consistent with our present findings, e.g., we found that rural-urban differences in obesity prevalence were not observed in overall Asian and subgroups except for Vietnamese, whose obesity rate was higher in urban than in rural areas. It is possible that rural-urban differences in obesity-related risk factors are different in Asians compared to other races, e.g., urban Asians are likely to have higher rates of health risk behaviors. In this study, urban Asians had higher percentage than rural Asians with regard to low education (i.e., high school degree, 30.1% vs. 22.4%, p = 0.0061) and poor family income (i.e., below 100% FPL, 15.3% vs.12.8%, p = 0.0003).

Despite some evidence for rural and urban differences in obesity using national data (e.g., NHANES, YRBSS, and NHIS), these surveys may ignore differences in Asian-American racial/ethnic groups^18,19^. The present study contributes to the limited literature regarding geographic disparities in obesity among Asian Americans. We found that Vietnamese living in urban areas had higher prevalence of obesity compared to those living in rural areas. Our study also shows that Japanese and Filipino are more likely to be obese compared to Chinese; it may be that the former two groups are more likely to be acculturated than Chinese, thus leading to similar lifestyle of U.S. native population. For example, English would be the mother language for Filipinos who are more likely to accept U.S. cultural lifestyle, e.g., consuming more fast food, etc. We also observed an inverse association between old age (i.e., 65 years or older) and obesity in rural areas. Many old people likely move to non-urban areas after their retirement, and live with healthier lifestyles.

Our study has some limitations. First, our data are subject to self-report bias. Self-reported height and weight used in this study likely underestimate obesity prevalence^20^ and may influence the degree to which obesity rates differ across rural and urban settings. A study^6^ shows that measured obesity rates were dramatically higher compared to self-reported estimates and were consistent with overall national obesity prevalence of 34% from NHANES 2007–2008^21^, highlighting the importance of using measured height and weight when determining population estimates. In addition, rural populations are older^22^ and appear to be heavier, and both of these factors are associated with inaccurate reporting of height and weight^23,24^. The analysis using objectively measured rural and urban obesity prevalence^6^ between Asian racial/ethnic groups are warranted in future studies. Second, the CHIS response rate was low, but lower response rates do not necessarily mean more biased estimates^25,26^. Studies comparing socio-demographics from CHIS and the California Census indicate that CHIS is representative of the California population. In addition, CHIS included a sample of cell phone-only households weighted to represent adults who only have a cell phone and do not have access to a landline telephone. Finally, it is a cross-sectional survey that cannot be used to establish temporality and, therefore, causality.

## Conclusion

Geography disparities were observed in obesity among Asians in California. Higher obesity prevalence was found in urban Vietnamese than in rural counterparts. Overall, younger age, being male, being Japanese, Filipino, or other Asians (vs. Chinese) were associated with higher prevalence of obesity. After being stratified by urban and nonurban areas, we also observed different number of risk factors of obesity among Asian Americans in California.
